# Age separation dramatically reduces COVID-19 mortality rate in a computational model of a large population

**DOI:** 10.1098/rsob.200213

**Published:** 2020-11-11

**Authors:** Liron Mizrahi, Huda Adwan Shekhidem, Shani Stern

**Affiliations:** Sagol Department of Neurobiology, Faculty of Natural Sciences, University of Haifa, Haifa 3498838, Israel

**Keywords:** COVID-19, corona virus, network, Erdős–Rényi model, computational model, age separation

## Abstract

COVID-19 pandemic has caused a global lockdown in many countries throughout the world. Faced with a new reality, and until a vaccine or efficient treatment is found, humanity must figure out ways to keep the economy going, on one hand, while keeping the population safe, on the other hand, especially those that are susceptible to this virus. Here, we use a Watts–Strogatz network simulation, with parameters that were drawn from what is already known about the virus, to explore five different scenarios of partial lockdown release in two geographical locations with different age distributions. We find that separating age groups by reducing interactions between them protects the general population and reduces mortality rates. Furthermore, the addition of new connections within the same age group to compensate for the lost connections outside the age group still has a strong beneficial influence and reduces the total death toll by about 62%. While complete isolation from society may be the most protective scenario for the elderly population, it would have an emotional and possibly cognitive impact that might outweigh its benefit. Therefore, we propose creating age-related social recommendations or even restrictions, thereby allowing social connections while still offering strong protection for the older population.

## 1. Introduction

The COVID-19 pandemic started in late December 2019 with mysterious pneumonia in Wuhan, China, and was declared a global pandemic by the World Health Organization (WHO) on 11 March 2020. The disease, caused by the SARS-CoV-2 pathogen, has quickly spread throughout 6 continents and over 210 countries. COVID-19 causes respiratory disease and is considered to be much more contagious than influenza [1]. Common symptoms include fever, dry cough, fatigue, shortness of breath and loss of smell or taste [2–5]. COVID-19-related complications include pneumonia and acute respiratory distress syndrome that may develop into a severe respiratory failure, septic shock and death [6,7]. In addition to being more contagious than influenza, COVID-19 has longer incubation periods compared with influenza. During the incubation period, the patients may be contagious [8–11]. Reports present incubation periods with a mean of 5–6 days, during which the patients are contagious [8,12]. Additionally, the mortality rate from COVID-19 disease is higher than the mortality rate from influenza complications [13]. These conditions instigated a rapid spread of the disease, causing over 100 countries to declare lockdowns and curfews, and causing an estimated global economic loss of one trillion US dollars in 2020 [14]. Till October 2020, over 36 million people were reported to be infected with COVID-19 and more than one million people died from virus-related complications.

The mortality rate from COVID-19 is strongly age biased, affecting the older population to a much greater extent [15–17]. In fact, it is thought that the younger population is usually asymptomatic or experience mild symptoms, even when infected with the virus [11,18]. For those symptomatic patients, the incubation period is the same regardless of their age. Recovery is reported to be 28 ± 14 days [19].

When the global lockdown is released, a slow release of the population back to their daily routine will occur. There is thought to be a psychological toll [20–22] due to the isolation of the population. Since COVID-19 is lethal mostly to the elderly population, suggestions of reopening the curfews, but keeping the elderly population isolated, have been proposed [23–25]. The psychological, emotional and even cognitive impacts may be stronger on this population, as social interactions are known to be essential for preventing a cognitive and physical decline [20,24,25]. Therefore, if there could be a solution where social interactions would not be prevented for the elderly population while keeping the population at low risk from the virus, this may be a preferred solution to this population.

The lower chances of the older population to be asymptomatic or to present mild symptoms compared with the younger population means that older COVID-19 patients are contagious for shorter periods of time compared with the younger patients. Asymptomatic (or weakly symptomatic) patients may be contagious all the way until full recovery (28 ± 14), whereas a symptomatic patient will be contagious for 6.4 ± 2.3 days (after which he will be isolated from society). This makes a young individual a stronger candidate for infecting others compared with an older individual. Here, we used these assumptions to derive a model of a large population to see what happens if we allow social interactions in the elderly population, but only among their own age group. This type of restriction will allow for the social interactions that are so important for the elderly age group. The model that we developed confirms that there would be a drastic reduction in mortality rate compared with allowing social interactions with other age groups (between 62% and 93%, depending on the scenario in a younger population distribution such as in Israel, and between 54% and 99%, depending on the scenario in an older population distribution such as in Italy), even when we keep the total number of connections the same by adding new connections within the elderly population for every lost connection with the younger population.

## 2. Methods

### 2.1. Population and network connectivity

**Figure 1.**
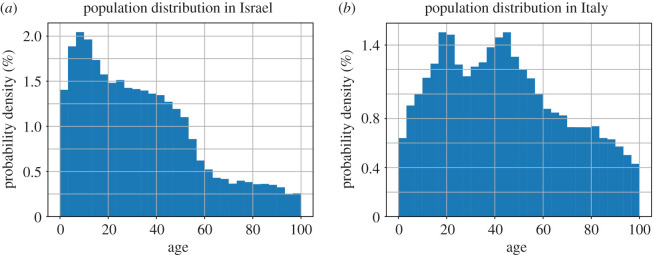
The distribution of the population by their age. (*a*) The population distribution in Israel. (*b*) The population distribution in Italy.

We simulated a population of 50 000 individuals using network theory and a Watts–Strogatz model network with a degree distribution of 15 [26]. The Watts–Strogatz model was chosen because this ‘small-world’ model captures the features of high clustering and has a small average number of degrees of separation between any two individuals, which have been widely observed in real-world networks. Additionally, we simulated an Erdős–Rényi model [27] since it is a simple random graph, easy to generate, with a fixed number of nodes. Each node has a similar number of connections (degree). However, random graphs lack some of the crucial properties of real social networks such as ‘clustering’, in which the probability of two people knowing one another is greatly increased if they have a common acquaintance. In a random graph, by contrast, the probability of there being a connection between any two people is uniform. The results with this model are presented in the electronic supplementary material for reference (electronic supplementary material, figures S1–S6). We separated the population into four age groups: 0–14, 15–34, 35–54 and 55+ years. We ran our models in two types of populations. One is a population that has a younger distribution. For this, the Israeli population distribution was chosen as presented by the Central Bureau of Statistics in Israel [28]. The second is an older distribution for which the Italian distribution was chosen as presented by the CIA World Factbook [29]. We chose the distributions as were published in the references mentioned and chose a normal distribution with an expectation of the middle point of the age group and a standard deviation of half of the range of that age group. The final distributions are presented in figure 1 and when integrating over all the age points in every age group, the percentages in each of the age group is similar to the one that we based it on [28] with up to 0.5% error. The derivation of the final distribution from the published distributions is presented in electronic supplementary material, figure S7A for the Israeli population and S7B for the Italian distribution.

**Figure 2.**
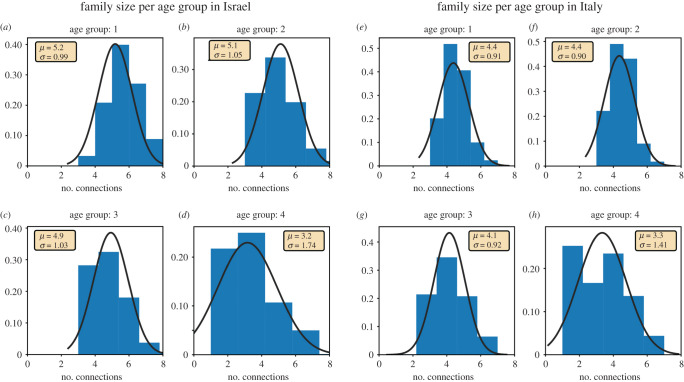
The distribution of family size by age group. (*a*) The family size distribution in age group 1 (0–14) in Israel. (*b*) The family size distribution in age group 2 (15–34) in Israel. (*c*) The family size distribution in age group 3 (35–54) in Israel. (*d*) The family size distribution in age group 3 (55+) in Israel. (*e*) The family size distribution in age group 1 (0–14) in Italy. (*f*) The family size distribution in age group 2 (15–34) in Italy. (*g*) The family size distribution in age group 3 (35–54) in Italy. (*h*) The family size distribution in age group 3 (55+) in Italy.

We chose the size of the families (or households) to be normally distributed with a mean and a standard deviation of 4 ± 1. Families were constructed in the following manner: After randomly grouping the entire population into families using a normal distribution with a mean of 4 and a standard deviation of 1, the family members were filled in according to the size of the family. If the family size was larger than 2, a pair of parents aged 20–60 were randomly taken from the population pool and the remaining members of the family were randomly selected with an age of 0–20. If the family had only two members, two adults with an age of 55–100 were randomly selected, and if the family size was 1, one adult with an age of 20–100 was randomly selected from the general population. These constraints resulted in the following family sizes according to the age groups: 5.2 ± 0.99 for the first age group, 5.1 ± 1.05 for the second group, 4.9 ± 1.03 for the third age group and 3.2 ± 1.74 for the fourth age group in the Israeli population. In the Italian population, this resulted in family sizes of 4.4 ± 0.91 for the first age group, 4.4 ± 0.90 for the second group, 4.1 ± 0.92 for the third age group and 3.3 ± 1.41 in the fourth age group. The populations' distributions are plotted for the different age groups in figure 2 (Israeli families on the left and Italian families on the right). The results for Erdős–Rényi model are similarly presented in electronic supplementary material, figure S1.

**Figure 3.**
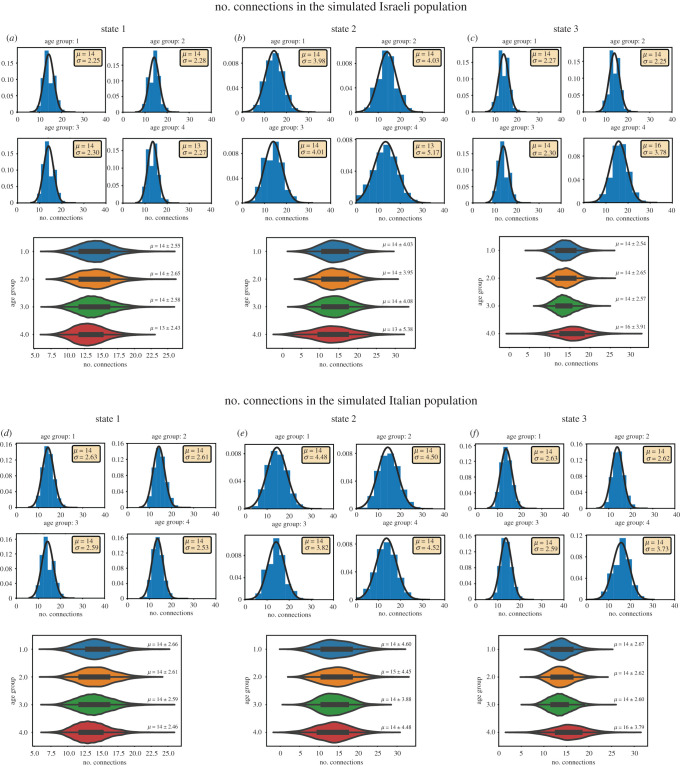
The distribution of the number of connections per individual in the different age groups in states 1–3 shows similar connectivity between these states. Simulations with an age distribution similar to the Israeli population are presented in (*a–c*), and similar to the Italian population is presented in (*d–f*). (*a*) The distribution of the connections in state 1 in the different age groups (Israel). (*b*) The distribution of the connections in state 2 in the different age groups (Israel). (*c*) The distribution of the connections in state 3 in the different age groups (Israel). (*d*) The distribution of the connections in state 1 in the different age groups (Italy). (*e*) The distribution of the connections in state 2 in the different age groups (Italy). (*f*) The distribution of the connections in state 3 in the different age groups (Italy). The number of connections did not change much between the states, indicating that disease evolution in these states changes mainly due to the age separation and not due to changes in the connectivity of the network.

**Figure 4.**
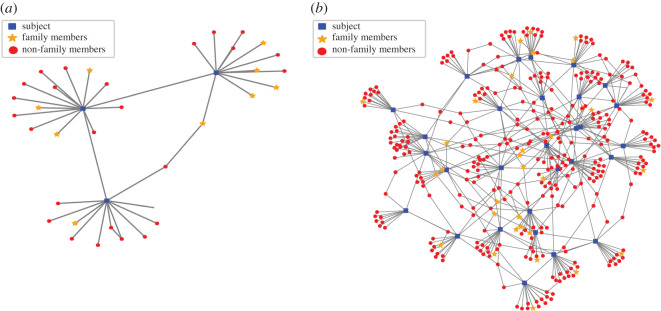
Example connections within a sample of the population. (*a*) Example of connections between 3 subjects. (*b*) Example connections between 30 subjects.

Due to the family members’ selection, the connectivity level in the different age groups also varied slightly and consisted of 14 ± 2.25 connections for the first age group, 14 ± 2.28 connections for the second age group, 14 ± 2.30 for the third age group and 13 ± 2.27 connections for the fourth age group in the Israeli population. Figure 3*a* shows the distribution of the number of connections according to the different age groups (these conditions will later be defined as state 1) in the Israeli population. Figure 3*d* similarly presents the number of connections in the Italian population showing a similar number of connections to the Israeli population. Figure 4 presents example connections within a general 50 000 individuals' population of three individuals (figure 4*a*) and of 30 individuals (figure 4*b*). Electronic supplementary material, figures S2 and S3 present the results for Erdős–Rényi model, respectively.

### 2.2. Infection

The model assumes different infection rates at different scenarios, but what is common is that once an individual becomes symptomatic, he is assumed to be under full quarantine and is therefore removed from the network. The highest infection rate occurs within the family and is 10% daily; each day each infected individual will infect another individual within his family with a probability of 10%. The infection rate in public is reduced to 1%, assuming people keep social distancing, thus reducing dramatically the infection rate.

Once infected, symptoms may appear in all age groups under a different probability. There is a higher probability to be asymptomatic (or presenting mild symptoms) for younger people. We used 80% chance to be asymptomatic for the 0–14 age group, 60% chance to be asymptomatic for the 15–34 age group, 40% chance for the 35–54 age group and 20% chance for the 55+ age group. These numbers were based on several reports [18,30,31]. For those patients that will become symptomatic, the number of days until presenting symptoms has a Weibull distribution with a mean of 6.4 days and standard deviation of 2.3 days [12], and this does not vary between the age groups.

Mortality rates vary dramatically among age groups and are 0% for 0–14, 0.15% for 15–34, 1% for 35–54 and 24% for ages 55+, similar to what was reported in [15]. Once showing symptoms the recovery rate (for those who recover) is 28 ± 14 days (normal distribution) in all age groups [19].

### 2.3. Creating multiple sample paths

We have run our model multiple times (*n* = 10) to achieve different sample paths in the model. This was done to get a better understanding of the average and the distribution of the results of a stochastic model such as the Watts–Strogatz. The algorithm for one infection simulation is described in electronic supplementary material, figure S4. Each run starts with a random and different five patients that are the first carriers. This allows for different realizations of the model and the results. To graphically show this, we added in figure 6 a plot that describes these 10 different instances by plotting the mean (solid line) and a 95% confidence interval for each of the states for a 250 days simulation run.

## 3. Results

We have defined five different states. The first state complies with all the above assumptions and with no other restrictions. Electronic supplementary material, videos 1 and 2 show the spread over time of COVID-19 demonstrating graphically the connections and infections in individuals in a population of 10 000 people for state 1 in Israel and Italy, respectively. Blue dots represent susceptible individuals, orange dots represent carrier individuals, red dots represent infected individuals and black dots are individuals who died from COVID-19. The shape of the dots marks the age groups (circle, 0–14; triangle, 15–34; square, 35–54; cross, 55+). We next wanted to test how age separation changes infection and mortality rates in the population. For this, we defined four more states with different scenarios of age separation. In state 2, we did not allow interactions between the different age groups, such that the interactions among family members remained without a change, but all other connections were eliminated. This reduced the connectivity of the network. Since we were interested to see the effect of the age separation and not the effect of reducing the connectivity, we added in these state new random connections within the same age group to replace any connection that was eliminated. The new number of connections for each group resembles the original conditions, and is shown in figure 3*b* for the Israeli population and in figure 3*e* for the Italian population (electronic supplementary material, figure S2 for the Israeli population using Erdős–Rényi model). The new number of connections for both populations is similar to the original number of connections (the family size distribution remains since we had not changed the family connections).

We next defined a more plausible constellation of the network. In state 3, we grouped the three age groups 0–14, 15–34 and 35–54, and did not allow connections with the elderly group of 55+ (except if they are in the same household). Such restrictions also reduced the overall number of connections, and we therefore added random connections within the same age group to any connection that was eliminated between the age groups. Figure 3*c* presents the new distribution of connections in the Israeli population per individual in the different age groups, and the number of connections is similar (or greater) than the original number of connections. Similarly, figure 3*f* presents the distribution of connections of state 3 in the Italian population, and there too the number of connections in state 3 is similar to the number of connections in state 1.

Since it is also reasonable to assume that restrictions of connections between age groups may reduce network connectivity, we added state 4 in which we keep all the connections, but reduce the connectivity between age groups by reducing by half the infection rates to 0.5% between age groups. We similarly defined state 5, where we completely diminish connections between different age groups (but without adding new connections).

**Figure 5.**
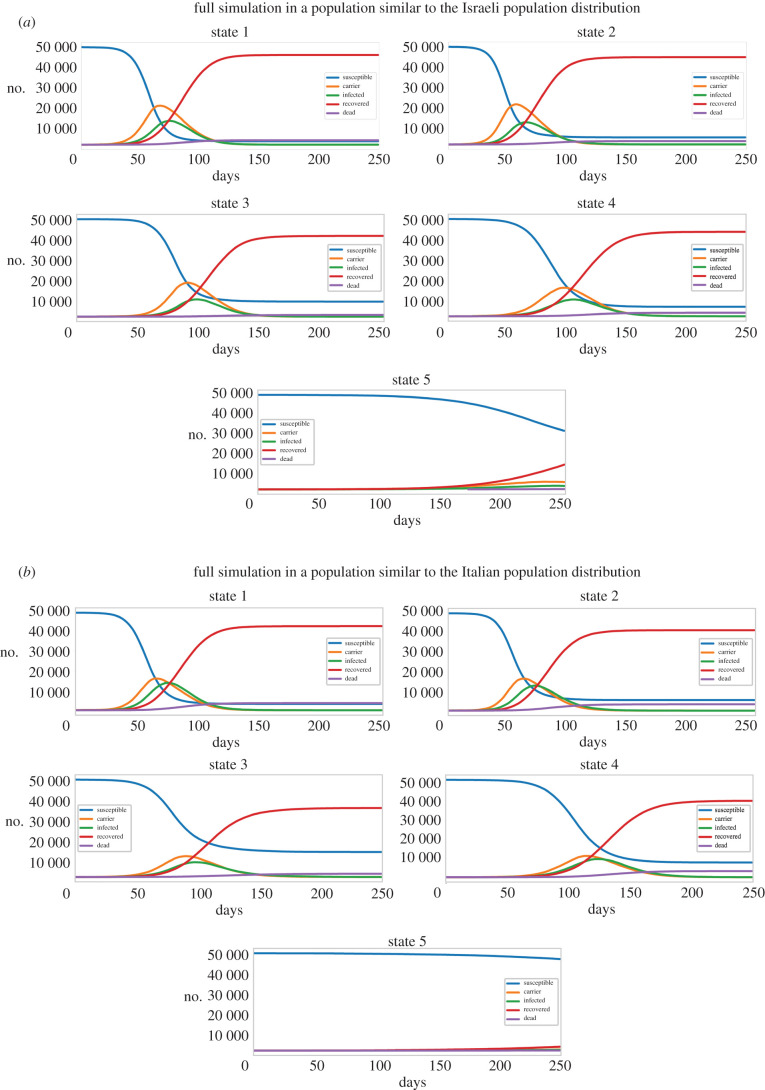
The total number of susceptible, carriers, infected, recovered and deceased individuals in the entire population of 50 000 people over a period of 250 days for states 1–5 for the Israeli population (*a*) and states 1–5 for the Italian population (*b*).

**Figure 6.**
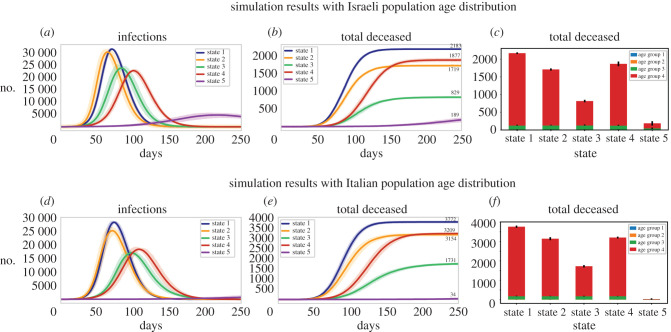
(*a*) A statistical span of the number of infected individuals in the entire population for the different states in Israel showing multiple realizations over *n* = 10 runs of the simulation for a better statistical average of the infection rates in the Israeli population (see Methods). (*b*) Similarly, a statistical span of the number of deceased individuals in the entire population in the different states in Israel (10 simulation runs). While in state 1 in a population distribution similar to the Israeli population 2183 individuals died on average, in state 2 only 1719 individuals died, in state 3 only 829 individuals died. In state 4, 1877 individuals died and in state 5 only 189 individuals died. (*c*) The distribution of deaths among the age groups in the different states for a population distribution similar to the Israeli distribution shows that the reduction in mortality occurs mainly due to the reduction in mortality rate in age group 4 and in state 5 also in age group 3 (but mostly in age group 4). (*d*) Similarly, a statistical span of the number of infected individuals in the entire population for the different states with a population with a distribution similar to Italy (10 simulation runs). (*e*) The total number of deceased in the entire population in the different states in Italy (10 simulation runs representing ten realizations). While in state 1 in a population distribution similar to the Italian population 3772 individuals died on average, in state 2 only 3154 individuals died, in state 3 only 1731 individuals died. In state 4, 3209 individuals died and in state 5 only 34 individuals died. (*f*) The distribution of deaths among the age groups in the different states for a population distribution similar to the Italian population distribution shows that the reduction in mortality occurs mainly due to the reduction in mortality rate in age group 4 and in state 5 also in age group 3.

Figure 5*a* presents the simulation results over a time period of 250 days in a population of 50 000 individuals (with statistics as in the Israeli population) by the categories of susceptible, carrier (has the virus), infected, recovered and deceased for the entire population pooled together in the five different states (electronic supplementary material, figure S5 presents results for the Erdős–Rényi model in the Israeli population). Similarly, figure 5*b* presents the simulation results over 50 000 individuals with statistics as in the Italian population. We expanded the plots to see in more detail the infection and death rates. The results are presented in figure 6*a–c* for the Israeli population and in figure 6*d–f* for the Italian population. For the Israeli population, figure 6*a* presents the infection over the 250 days for the five different states. Figure 6*b* similarly shows mortality from the virus over the course of 250 days in the different states. Further comparison of infection and mortality rates (figure 6*a,b*, respectively) shows dramatic infection rate changes, and most importantly lower mortality in the different states (2–5) compared with state 1. This shows that reducing the interaction between age groups decreases mortality, even when keeping the number of interactions between individuals constant or even increasing this number. Separating just the older group (55+) from interacting with the other age groups (but keeping interactions within the same household, state 3) reduces the overall mortality rate in the population by 62%. This decrease becomes even more pronounced when not adding new connections within the same age group (state 5) with a reduction of 93% in the mortality rate in the overall population. We plotted also the mortality rate in each of the states in the different age groups (figure 6*c*) for the Israeli population. The plots show that most of the decrease in mortality rate occurs due to a decrease in the mortality of the oldest age group and in state 5 also of the second oldest age group (but to a lower extent). The results for the Italian population are presented in figure 6*d,e* and a reduction of 54% can be observed in state 3 and of 99% in state 5. Similarly, figure 6*f* shows that most of the reduction in mortality rate is due to a reduction in the mortality of the oldest age group. Using this strategy in a real-world scenario would probably result in a reduction that is somewhere between these two states (state 3 and state 5) since some interactions will be replaced (such as seats in the theatre), but some will be eliminated (such as meeting distant relatives). The results using Erdős–Rényi model are presented in electronic supplementary material, figure S6.

Electronic supplementary material, videos S3 and S4 present the spread of the disease in states 1–5 in Israel and Italy, respectively. The colour and shape schemes are the same as in electronic supplementary material, videos S1 and S2. Electronic supplementary material, videos S5 and S6 similarly show the same disease evolution as electronic supplementary material videos S3 and S4, but the blue dots were removed for clearer graphs. A clear reduction in the death rate is exhibited in the new states.

## 4. Discussion

COVID-19 caught the world unprepared and has infected (by October 2020) over 36 million people (reported) and has cost over one million lives. The pandemic has caused a global lockdown that in turn caused a huge economic burden. Releasing the lockdown needs to be under controlled conditions, being extremely careful. Currently, most countries are in the ‘second wave’ [32,33] and we are still far from resolving this pandemic. The elderly population are the most susceptible to this virus, and therefore many suggestions have been made of releasing the general population, while keeping the elderly quarantined. However, social interactions are known to be extremely important in the elderly population [34,35], and such conditions may backfire leading to both mental and emotional deterioration in this population.

In this study, we tested several states that limit the interactions between people in different age groups, and we showed that all these scenarios reduce mortality rate. In the first two states, we kept the interactions of family members within the same household, and eliminated connections outside of the age group, yet keeping the network connectivity (by adding more connections within the same age group). This drastically reduces the overall death toll by 62%. The reasoning for the improvement in the overall mortality is that younger people have a higher probability of being asymptomatic (or weekly symptomatic). Asymptomatic people usually keep on their social interactions, infecting many people for a very long time. Older people have a much lower probability of being asymptomatic. This means that they are usually infectious for only approximately 5–6 days (the asymptomatic period is assumed to have a Weibull distribution with an average of 6.4 days). At this point, they usually know that they are sick and are isolated. Therefore, an older individual is less likely to spread the disease.

In the other two states, we reduced the interactions (state 4) or completely diminished the interactions (state 5) between the different age groups. This may be a reasonable assumption, since not all connections between age groups will be replaced by connections within the age group (such as connections with distant family). In state 5, all the connections outside the age group were eliminated, which reduced mortality rate by 93% in a population with an Israeli distribution (99% in a population with an Italian distribution). A real scenario will probably be some compromise between state 3 and state 5, since some connections will indeed be replaced (for example, seating in a restaurant) and some will be lost (such as meeting distant family members). It is important to keep in mind that our model is a stochastic model that depends on the initial conditions. To better understand how variable the results are with respect to different realizations or sample paths, we ran the model 10 times and plotted in figure 6 the confidence interval around the mean total infected and deceased individuals for each of the conditions.

It is important to note that although age separation is difficult to implement generally, it is relatively easy to find microenvironments in which age separation is possible and will enable the older population to maintain social connections while reducing infection and death rates in these populations. Such microenvironments may include grocery shops, theatres or airplane rides, and would allow important social interactions for the elderly population. Overall, our study shows that age separation is extremely beneficial and can be imposed in an important intermediate period until resuming normal life when finding a cure or a vaccine to COVID-19.

## Supplementary Material

Supplementary Video 1

## Supplementary Material

Supplementary Video 2

## Supplementary Material

Supplementary Video 3

## Supplementary Material

Supplementary Video 4

## Supplementary Material

Supplementary Video 5

## Supplementary Material

Supplementary Video 6

## Supplementary Material

Supplementary Material

## Data accessibility

The source code for our study can be found in the following link: https://github.com/Precision-Disease-Modeling-Lab/Covid19-Computational-Model.git.
