# Supplementary material for: Age separation dramatically reduces COVID-19 mortality rate in a computational model of a large population
Source: Open Biol. 2020 Nov 11;10(11):200213. doi: 10.1098/rsob.200213 (PMC7729024; doi:10.1098/rsob.200213)
Supplement: Supplementary Material [file rsob200213supp7.docx]

**Supplementary Material**

**Supplementary Figure 1**

**
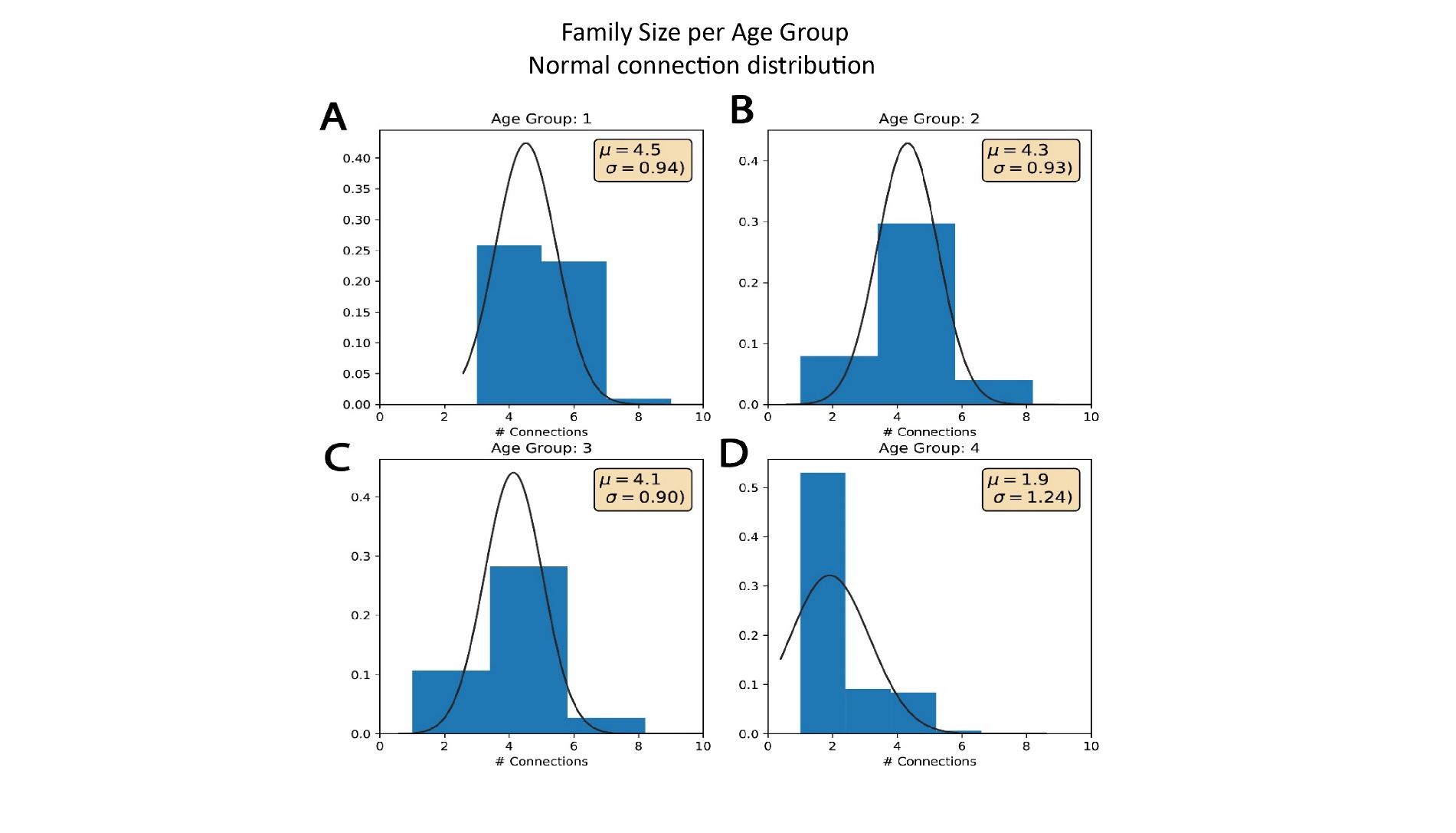
**

**Supplementary Figure 1.** The distribution of family size by age group using Erdős-Rényi model. A. The family size distribution in age group 1 (0-14). B. The family size distribution in age group 2 (15-34). C. The family size distribution in age group 3 (35-54). D. The family size distribution in age group 4 (55+).

**Supplementary Figure 2**


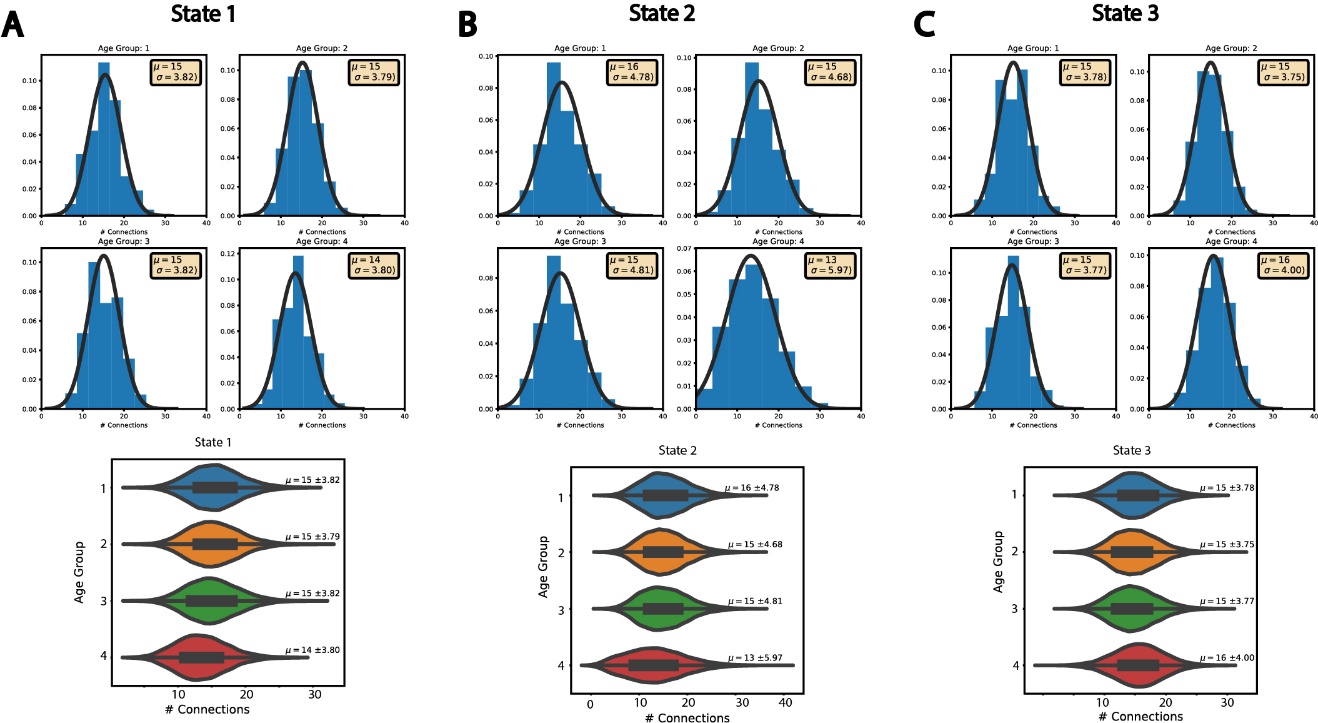


**Supplementary Figure 2.** The distribution of the number of connections per individual in the different age groups in states 1-3 shows similar connectivity using Erdős-Rényi model. A. The distribution of the connections in state 1 in the different age groups. B. The distribution of the connections in state 2 in the different age groups. C. The distribution of connections in state 3 in the different age groups. The number of connections using Erdős-Rényi model did not change much between the states, indicating that disease evolution in these states changes mainly due to the age separation and not changes in the connectivity of the network.

**Supplementary Figure 3**

**
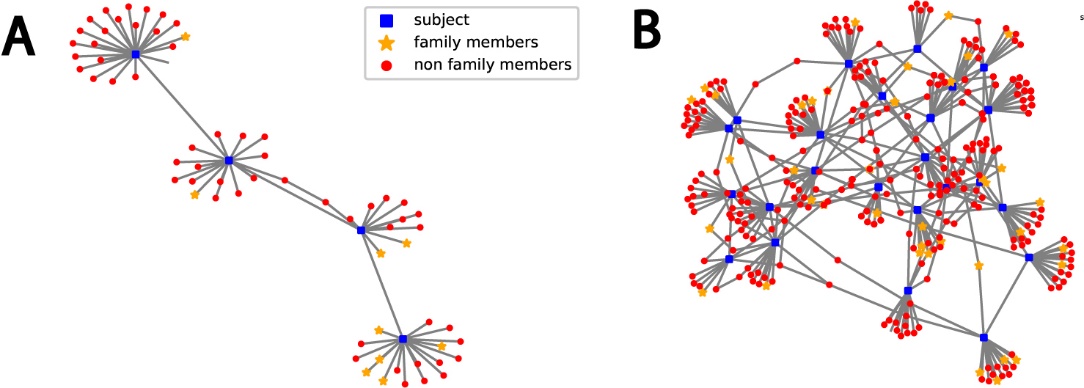
**

**Supplementary Figure 3.** Example connections within the population using Erdős-Rényi model. A. Example of connections between 3 subjects. B. Example of connections between 30 subjects.

**Supplementary Figure 4**

**
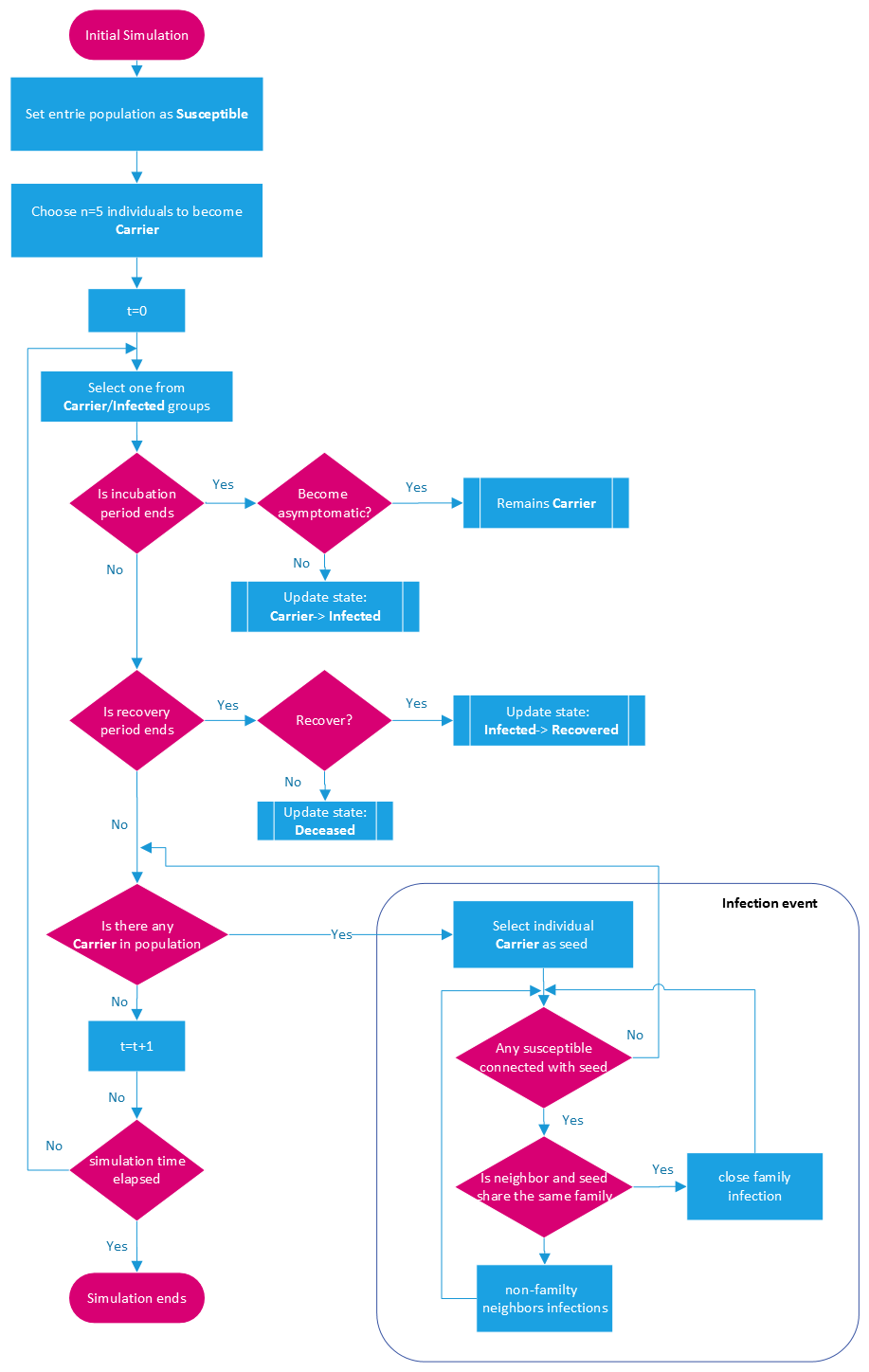
**

**Supplementary Figure 4.** A block diagram describing the algorithm for the simulation analysis (used for both Watts–Strogatz and Erdős-Rényi model)

**Supplementary Figure 5**

**
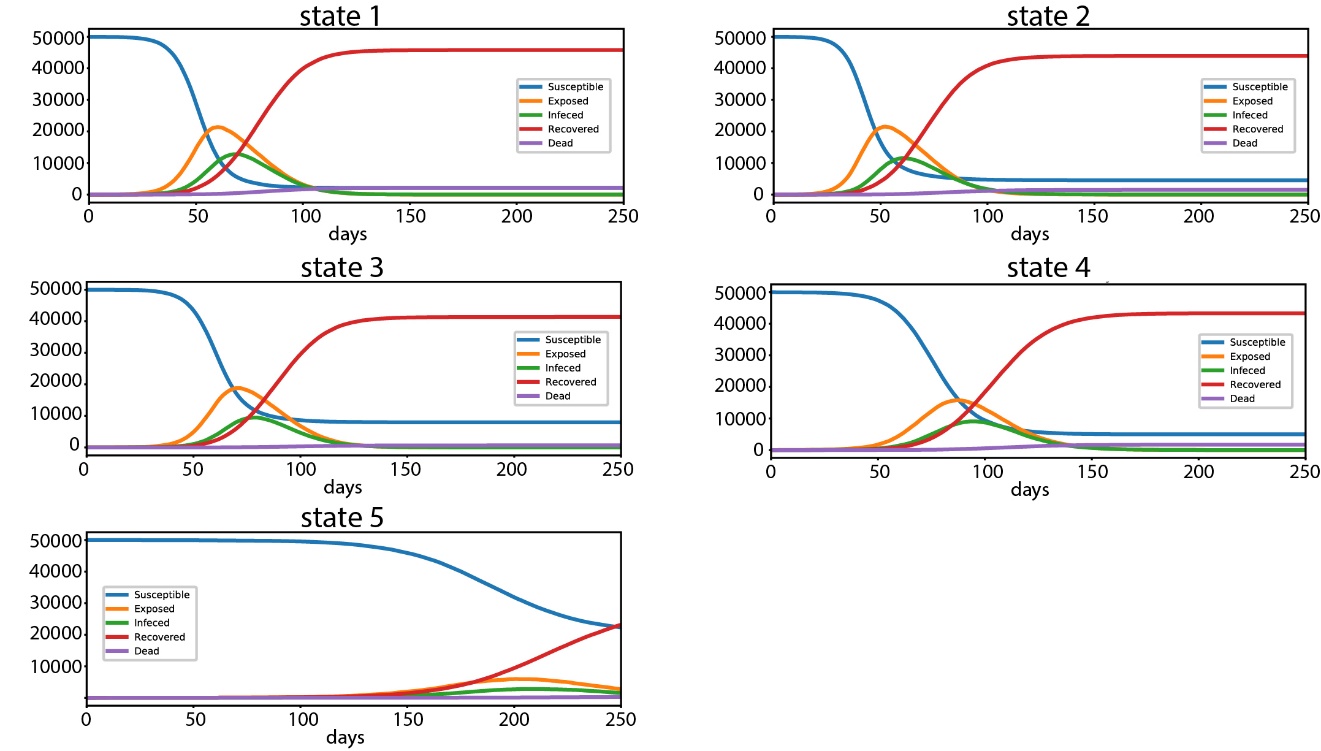
**

**Supplementary Figure 5.** The total number of susceptible, carriers, infected, recovered and deceased individuals in the entire population of 50000 people over a period of 250 days in the different states. A-E. For state1-state5 respectively.

**Supplementary Figure 6**

**
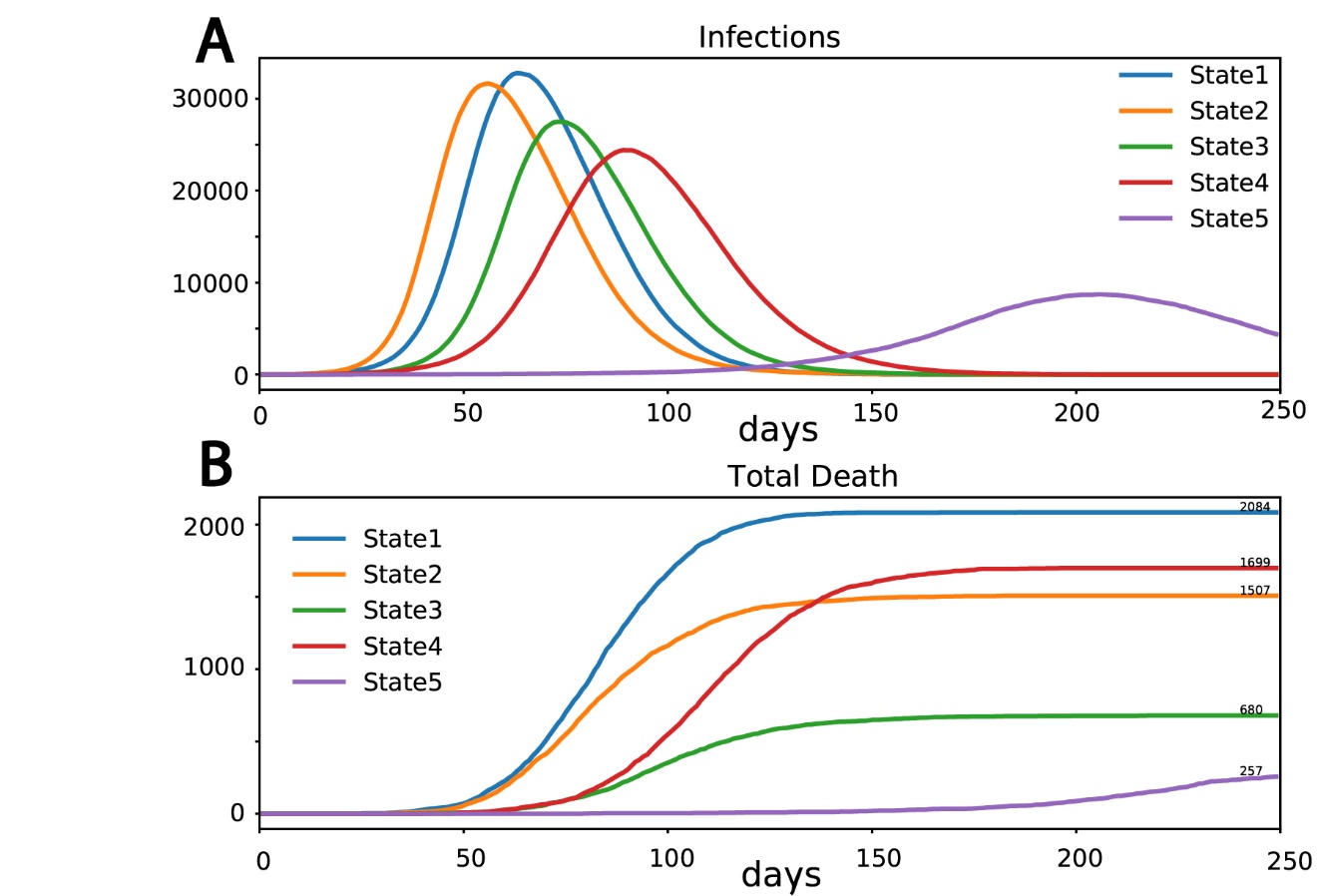
**

**Supplementary Figure 6.** A. Total number of infections in the entire population for the different states. B. Total number of deceased in the entire population in the different states. While in state 1 2084 individuals died, in state 2 only 1507 individuals died, in state 3 only 680 individuals died. In state 4 1699 individuals died and in state 5 only 257 individuals died.

**Supplementary Figure 7**

**
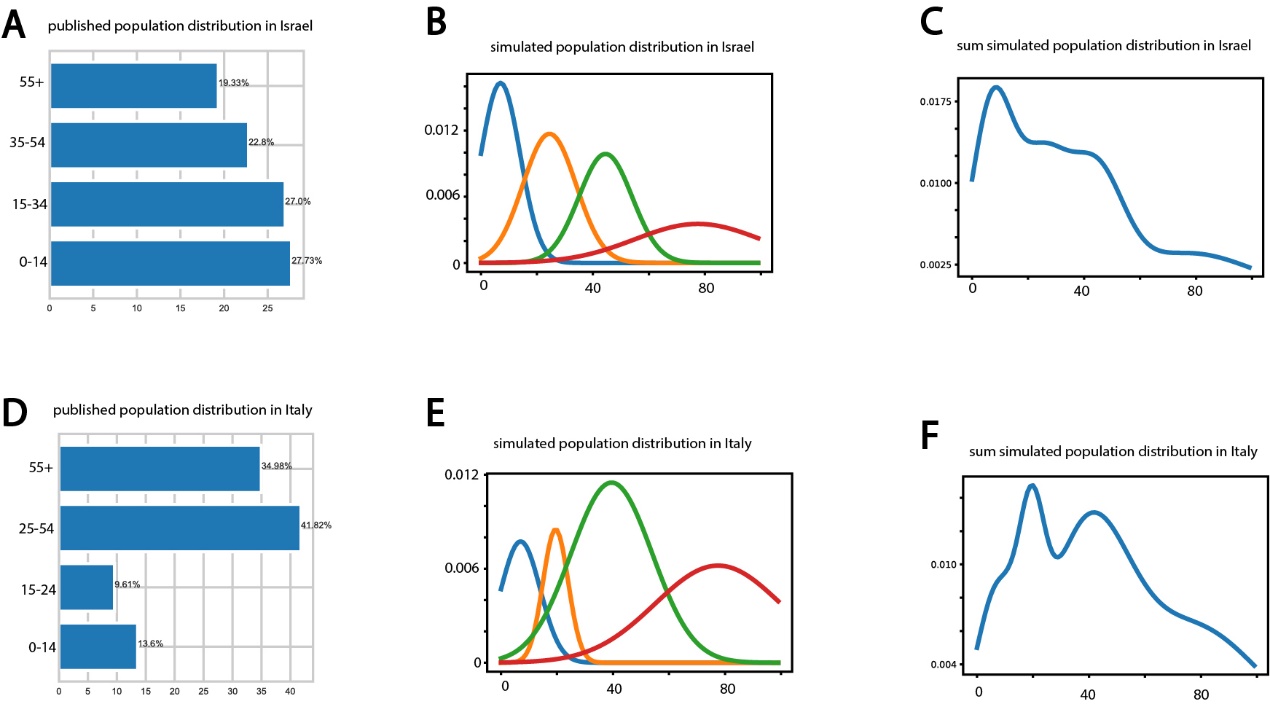
**

**Supplementary Figure 7.** Population distributions by age groups. A-C. Israeli distribution. B-D. Italian distribution. A. Our final distribution was derived from the published distribution presented (Israel). B. We generated 4 Normal distributions with a mean of the middle age range of each of the groups and a standard deviation that is half of the range of the ages in that age group (Israel). C. The sum of the Normal distributions in B is the final Israeli population distribution that was used for the simulation. D. Our final distribution was derived from the published distribution presented (Italy). E. We generated 4 Normal distributions with a mean of the middle age range of each of the groups and a standard deviation that is half of the range of the ages in that age group (Italy). F. The sum of the Normal distributions in E is the final Italian population distribution that was used for the simulation. The final distributions deviate by less than 0.5% from the original distribution taken from published statistics in each age group (1, 2).

**Supplementary Video 1.** A video demonstrating the spread of COVID-19 throughout a 10,000 individuals’ population for state 1 in the Israeli population. Blue are susceptible individuals, orange are carrier individuals, red are infected individuals and black are deceased. The first age group (0-14) is marked in circles, the second age group (15-34) is marked in triangles, the third age group (35-54) is marked in squares, and the fourth age group (55+) is marked in x. The video shows the evolution of the disease in state 1.

**Supplementary Video 2.** A video demonstrating the spread of COVID-19 throughout a 10,000 individuals’ population for state 1 in the Italian population. Blue are susceptible individuals, orange are carrier individuals, red are infected individuals and black are deceased. The first age group (0-14) is marked in circles, the second age group (15-34) is marked in triangles, the third age group (35-54) is marked in squares, and the fourth age group (55+) is marked in x. The video shows the evolution of the disease in state 1.

**Supplementary Video 3.** A video demonstrating the spread of COVID-19 throughout a 10,000 individuals’ population for states 1-5 in the Israeli population. Blue are susceptible individuals, orange are carrier individuals, red are infected individuals and black are deceased. The first age group (0-14) is marked in circles, the second age group (15-34) is marked in triangles, the third age group (35-54) is marked in squares, and the fourth age group (55+) is marked in x.

**Supplementary Video 4.** A video demonstrating the spread of COVID-19 throughout a 10,000 individuals’ population for states 1-5 in the Italian population. Blue are susceptible individuals, orange are carrier individuals, red are infected individuals and black are deceased. The first age group (0-14) is marked in circles, the second age group (15-34) is marked in triangles, the third age group (35-54) is marked in squares, and the fourth age group (55+) is marked in x.

**Supplementary Video 5.** Presenting the same conditions as Supplemental video 3, but the blue dots have been removed for a clearer observation.

**Supplementary Video 6.** Presenting the same conditions as Supplemental video 4, but the blue dots have been removed for a clearer observation.

1. facebood CW. Italy Age structure. index mundi. 2018.

2. The central Bureau of statistics I. אוכלוסייה, לפי קבוצת אוכלוסייה,

דת, גיל ומין, מחוז ונפה. למ''ס, הלשכה המרכזית לסטטיסטיקה. 2017.
